# Risk factors of dermatophytosis among Korean adults

**DOI:** 10.1038/s41598-022-17744-5

**Published:** 2022-08-04

**Authors:** Joon Ho Son, Jee Yun Doh, Kyungdo Han, Yeong Ho Kim, Ju Hee Han, Chul Hwan Bang, Young Min Park, Ji Hyun Lee

**Affiliations:** 1 Department of Dermatology, Seoul St. Mary’s Hospital, College of Medicine, The Catholic University of Korea, 222, Banpo-daero, Seocho-gu, Seoul, 06591 Republic of Korea; 2 Department of Statistics and Actuarial Science, Soongsil University, Seoul, Republic of Korea

**Keywords:** Diseases, Risk factors

## Abstract

Dermatophytosis includes all fungal infections caused by dermatophytes in humans. Some risk factors for the development of subtypes of dermatophytosis have been studied; however, large-scale epidemiologic studies on risk factors for total dermatophytosis are scarce. We investigated the risk factors of dermatophytosis using a nationwide study. Total 4,532,655 subjects with dermatophytosis aged between 20 and 40 years were examined using data from the Korean National Health Insurance Service from 2009 to 2018. Women showed a lower risk of development of dermatophytosis compared to men [hazard ratio (HR) 0.848; 95% confidence interval (CI) 0.843–0.853]. Subjects with elevated waist circumference (HR 1.057; 95% CI 1.048–1.065), heavy drinking (HR 1.053; 95% CI 1044–1.061), engaging in mild-to-heavy exercise (HR 1.071; 95% CI 1.064–1.077) had a higher risk of dermatophytosis. In addition, subjects with body mass index (BMI) of more than 30 kg/m^2^ exhibited a higher risk of dermatophytosis (HR 1.36; 95% CI 1.342–1.378) compared to those with BMIs in the range of 18.5–23 kg/m^2^. In this study, the risk of developing dermatophytosis significantly increased in individuals with elevated waist circumference or high BMI. Lifestyle modifications, including weight management, are suggested to be important in preventing dermatophytosis.

## Introduction

Fungal infections are an important public health concern due to their high prevalence worldwide^1^. Among them, dermatophytosis infections caused by dermatophytes are the most common cause of fungal infection in humans, affecting 20–25% of the world’s population^2^. Dermatophytosis, also referred to as tinea or ringworm, shows variable clinical manifestations and are usually classified according to the site of infection^1,3^. Tinea pedis and tinea unguim are the most common types of dermatophytosis, which are usually caused by *Trichophyton rubrum* and *T. interdigitale*, whereas tinea capitis is often casued by *Microsporum canis, T. tonsurans*, and *T. violaceum*^1,3–5^.

Several risk factors for dermatophytosis have been discussed. Host factors such as age, sex, and race have been found commonly to be significant in the development of infection^1,3,6^. However, while there are common characteristics shared between different types of dermatophytosis, there also exist differences in the characteristics, including risk factors, of each type^3^. Furthermore, the distribution of dermatophytosis, the etiological agents, and the predominant anatomical infection patterns vary with geographical location, culture, and environment^1,3,7^. For example, tinea pedis and onychomycosis, the most common types of dermatophytosis worldwide, are more prevalent in developed countries, whereas tinea capitis is more prevalent in developing countries^1^. However, in a study of the prevalence of skin infections in Libya, tinea corporis was the most common mycotic infection, accounting for 45.9% of cases, while tinea pedis only accounted for 8.1% of cases^8^.

Since studies on the risk factors of dermatophytosis remain limited in number, through a nationwide large-scale investigation, we identified epidemiologic risk factors of dermatophytosis in young adults of Korea using the database established by the Korean National Health Insurance Service (KNHIS).

## Materials and methods

### Data source

We obtained data from the national health claims database established by the KNHIS. The KNHIS system provides comprehensive medical care covering the entire Korean population and maintains a database of inpatient and outpatient medical records, including diagnostic and procedural codes, demographic data, and prescribed medication claims. The health care records of all Korean nationals are considered highly reliable as health records are tracked using a unique identification number each individual receives at birth, and the KNHIS follows the standard codes of the International Statistical Classification of Diseases and Related Health Conditions, 10th revision (ICD-10). The KNHIS also provides a free general health check-up program and a cancer pre-screening program at various stages of life. All subjects registered in the KNHIS database are advised to undergo national health check-ups every 2 years. The categories of health check-ups include anthropometric data, blood pressure, and laboratory data, such as fasting glucose levels, cholesterol levels, and serum creatinine levels. Using standardized self-reporting questionnaires, data on previous medical history and lifestyle factors, including smoking habits, alcohol consumption, and physical activity, were collected. Therefore, comprehensive information is available on all insured Koreans and their dependents concerning their various health exam results at different ages.

### Study design and population

**Figure 1:**
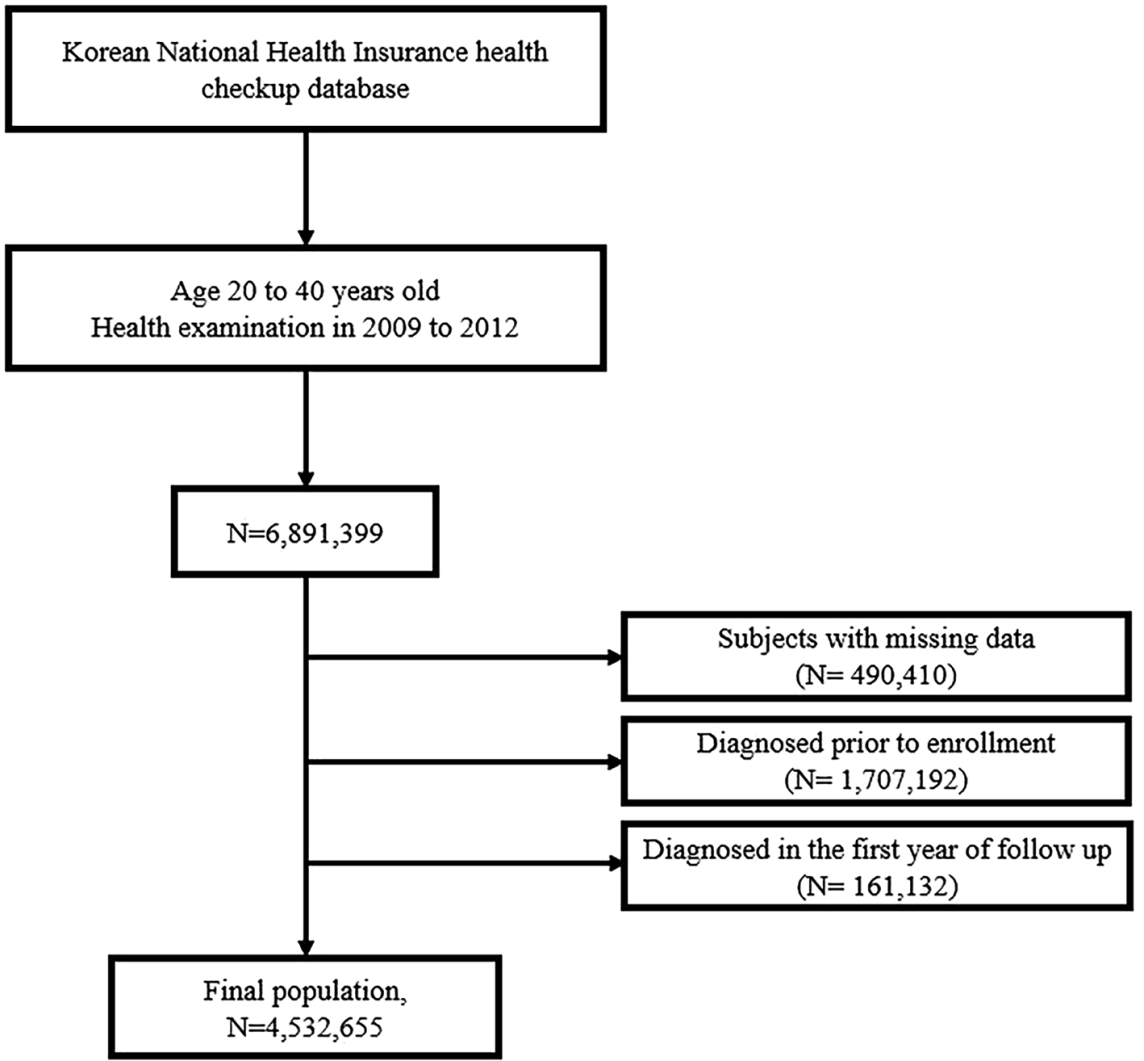
Study population: data from the Korean National Health Insurance Service.

This study was designed based on a retrospective analysis using the KNHIS, and we conducted a nationwide population-based longitudinal cohort study. Data of all civilians aged 20–40 years at the time of their medical examination (*N* = 6,891,399) who had undergone health examinations between 2009 and 2012 were abstracted from the KNHIS database. The disease codes retrieved included the following: dermatophytosis (ICD-B35), tinea barbae and tinea capitis (ICD-B35.0), tinea unguium (ICD-B35.1), tinea manuum (ICD-B35.2), tinea pedis (ICD-B35.3), tinea corporis (ICD-B35.4), tinea imbricata (ICD-B35.5), tinea cruris (ICD-B35.6), other type of dermatophytosis (ICD-B35.8), and dermatophytosis, unspecified (ICD-B35.9). Any subjects with missing data (*n* = 490,410), diagnosed with dermatophytosis prior to enrollment (*n* = 1,707,192), or who developed dermatophytosis in the first year of follow-up (*n* = 161,132) were excluded. A total of 4,532,655 subjects were finally included in this study (Fig. 1). Medical and health records of selected subjects were tracked until December 31, 2018, to identify patients with newly developed dermatophytosis.

### Clinical, laboratory, and anthropometric measurements

Data on sex, age, household income, alcohol consumption, smoking status, physical activity, comorbidities, waist circumference, body mass index (BMI), and lipid profiles were collected for the study. Demographic data of sex, age, and household income which was dichotomized at lower 20% (≤ 20% vs. > 20%)—were obtained from the results of the regular medical check-up programs of the KNHIS. Detailed histories about alcohol consumption, smoking status, and physical activity, including the amount and frequency, were obtained via a questionnaire given during the KNHIS health check-up programs. In this study, smoking status was classified as non-smoker, ex-smoker, or current smoker, and alcohol consumption was classified as abstinence (no alcoholic drinks consumed within the last year), moderate drinking (< 30 g of pure alcohol per day), or heavy drinking (≥ 30 g of pure alcohol per day). Physical activity was classified as none or moderate-to-severe. Moderate-to-severe physical activity was defined as 5 days or more of moderate-intensity exercise per week or 3 days or more of vigorous-intensity exercise per week. A history of comorbidities, including hypertension, dyslipidemia, and diabetes mellitus, was identified based on the combination of ICD-10 clinic and pharmacy codes and a list of prescribed medicines. Venous blood samples for measuring lipid profiles were collected after an overnight fast. Waist circumference was classified based on a cutoff point of 90 cm for men and 85 cm for women; values higher than these are considered to indicate abdominal obesity in Korean according to the Korean Society for the Study of Obesity. BMI was calculated by dividing each participant’s weight (in kilograms) by the square value of their height (in meters), which was measured during the check-up programs. BMIs were classified as underweight (< 18.5 kg/m^2^), normal (18.5–22.9 kg/m^2^), overweight (23–24.9 kg/m^2^), obese (25–29.9 kg/m^2^), or severely obese (≥ 30 kg/m^2^). All clinical, laboratory, and anthropometric measurement data used in the study was based on medical examination performed between 2009 and 2012.

### Statistical analysis and ethics statement

Baseline demographic characteristics of the study population are described using numbers and percentages or mean ± standard deviation values. An independent t-test for continuous variables and a chi-squared test for categorical variables, respectively, were used to analyze the differences in characteristics between the dermatophytosis group and non-dermatophytosis group. To evaluate the risk of development of dermatophytosis, Cox proportional hazards regression analyses were performed; then, multivariate Cox proportional hazard regression analyses were performed with variables showing a *P-*value of less than 0.05 in the univariate analysis used to determine the association between suspected risk factors and the prospective development of dermatophytosis. Lastly, cumulative incidence rates of dermatophytosis for the follow-up period were analyzed using the Kaplan–Meier method, and log-rank tests were performed to analyze the differences in the incidence of dermatophytosis between the BMI groups adjusted for other confounding variables, such as sex, age, alcohol consumption, smoking status, physical activity, household income, diabetes mellitus, hypertension, and dyslipidemia. All the statistical analyses were performed using the SAS software program (version 9.4; SAS Institute, Cary, NC, USA), and *P*-values less than 0.05 were considered to be statistically significant. This study was approved by the ethics committee of Seoul St. Mary's Hospital, the Catholic University of Korea (IRB no. KC21ZISI0279), and was conducted in accordance with the principles of the Declaration of Helsinki.

### Ethics declarations

We confirm that all methods were carried out in accordance with relevant guidelines and regulations.

### Institutional Review Board (IRB) approval status

Reviewed and approved by the IRB of the Catholic University of Korea (IRB approval no. KC21ZISI0279).

### Informed consent and permission

Exempted by the IRB of the Catholic University of Korea (IRB approval no. KC21ZISI0279) since this study only used anonymized medical records.

## Results

### Characteristics of the study population

During the study period, 861,840 dermatophytosis cases occurred among the 4,532,665 study subjects. Table 1 shows the characteristics of the study population, comparing the dermatophytosis and the non-dermatophytosis groups. Both groups showed statistically significant differences (*P* < 0.001) in all of the variables collected for this study. The dermatophytosis group tended to be older (30.86 vs. 30.41 years; *P* < 0.001) and had greater proportions of men (62.59% vs. 55.56%; *P* < 0.001), heavy drinkers (9.34% vs. 8.37%; *P* < 0.001), current smokers (36.44% vs. 34.75%; *P* < 0.001), ex-smokers (11.49% vs. 9.53%; *P* < 0.001), and those engaging in moderate-to-intense physical activity (13.46% vs. 11.95%; *P* < 0.001). The dermatophytosis group also had a smaller portion of those with low income compared to the non-dermatophytosis group (14.72% vs. 17.09%; *P* < 0.001) and exhibited a greater waist circumference (14.21% vs. 11.08%; *P* < 0.001) and higher prevalence rates of diabetes (1.99% vs. 1.74%; *P* < 0.001), dyslipidemia (7.08% vs. 6.33%; *P* < 0.001), and hypertension (7.59% vs. 6.83%; *P* < 0.001) compared to the non-dermatophytosis group.

**Table 1:** Characteristics of the study population.

	Dermatophytosis	Non-dermatophytosis	*P*
	(*n* = 861,840)	(*n* = 3,670,825)	
Age (years)	30.86 ± 4.99	30.41 ± 5.02	< 0.001
< 30 (%)	361,299 (41.92)	1,690,251 (46.04)	< 0.001
≥ 30 (%)	500,541 (58.08)	1,980,610 (53.96)	
Sex			< 0.001
Male	539,449 (62.59)	2,039,598 (55.56)	
Female	322,391 (37.41)	1,631,227 (44.44)	
Drinking			< 0.001
None (%)	311,253 (36.11)	1,428,335 (38.91)	
Mild (%)	470,110 (54.55)	1,935,192(52.72)	
Heavy (%)	80,477 (9.34)	307,298 (8.37)	
Smoking			< 0.001
Non-smoker (%)	448,755 (52.07)	2,525,638 (55.72)	
Ex-smoker (%)	98,991(11.49)	431,999 (9.53)	
Current smoker (%)	314,094 (36.44)	1,575,028 (34.75)	
Exercise (%)	116,026 (13.46)	438,667 (11.95)	< 0.001
Low income (%)^a^	126,846 (14.72)	627,389 (17.09)	< 0.001
Comorbidities			
Elevated waist circumference^b^	122,481 (14.21)	406,768 (11.08)	< 0.001
Diabetes	17,161 (1.99)	63,961 (1.74)	< 0.001
Dyslipidemia	61,002 (7.08)	232,439 (6.33)	< 0.001
Hypertension	65,390 (7.59)	250,541 (6.83)	< 0.001
Systolic pressure (mmHg)	118.05 ± 13.12	117.22 ± 13.22	< 0.001
Diastolic pressure (mmHg)	73.99 ± 9.43	73.46 ± 9.44	< 0.001
HDL-cholesterol (mg/dL)	57.18 ± 26.72	58.11 ± 27.35	< 0.001
LDL-cholesterol (mg/dL)	114.68 ± 222.4	113.98 ± 261.53	< 0.001

Values are expressed as mean ± standard deviation or n (%) by independent t-test (continuous variables) or chi-squared test (categorical variables).

*HDL* high-density lipoprotein, *LDL* low-density lipoprotein.

^a^Low income is defined as a household income of 20% or less of the median.

^b^Elevated waist circumference is defined as a waist circumference of more than 90 cm in men or more than 85 cm in women.

### Analysis for suspected risk factors of dermatophytosis

Table 2 shows the results of univariate and multivariate analyses. The results of the univariate analysis indicated that sex, age, BMI, waist circumference, drinking status, and exercise status were associated with an increased risk of developing dermatophytosis. Women were found to be less likely to develop dermatophytosis than men (hazard ratio [HR] 0.78; 95% confidence interval [CI] 0.777–0.784). Dermatophytosis developed more commonly in individuals older than 30 years of age (HR 1.137; 95% CI 1.132–1.142), those with a greater waist circumference (HR 1.296; 95% CI 1.288–1.304), and those who engage in moderate-to-heavy exercise (HR 1.126; 95% CI 1.119–1.133). Dermatophytosis also appeared to favor heavy drinkers (HR 1.099; 95% CI 1.095–1.104 for mild drinkers vs. HR 1.171; 95% CI 1.162–1.18 for heavy drinkers). Regarding the BMI status, the HRs of dermatophytosis incidence showed an increasing trend as the obese status was aggravated, while those who were underweight (BMI < 18.5 kg/m^2^) showed a lower incidence of the development of dermatophytosis (HR 0.836; 95% CI 0.829–0.843 for those with BMIs < 18.5 kg/m^2^ vs. HR 1.207; 95% CI 1.201–1.214 for those with BMIs of 23–24.9 kg/m^2^ vs. HR 1.35; 95% CI 1.342–1.357 for those with BMIs of 25–29.9 kg/m^2^ vs. HR 1.436; 95% CI 1.422–1.451 for those with BMIs ≥ 30 kg/m^2^).

**Table 2:** Association between suspected risk factors and the incidence of dermatophytosis.

	Cases	Person-years	Incidence rate^a^	HR (95% CI)	
				Univariate analysis	Multivariate analysis
**Sex**					
Male	539,449	16,924,327.78	31.8742	1	1
Female	322,391	12,956,197.39	24.8831	0.78 (0.777–0.784)	0.848 (0.843–0.853)
**Age**					
< 30	361,299	13,458,176.72	26.8461	1	1
≥ 30	500,541	16,422,348.45	30.4793	1.137 (1.132–1.142)	1.064 (1.059–1.068)
**BMI (kg/m**^**2**^**)**					
< 18.5	388,377	2,616,765.9	21.7329	0.836 (0.829–0.843)	0.876 (0.868–0.884)
18.5–22.9	2,223,440	14,804,553.51	26.0108	1	1
23–24.9	837,991	5,493,166.73	31.4218	1.207 (1.201–1.214)	1.153 (1.146–1.159)
25–29.9	814,417	2,904,629.43	35.1455	1.35 (1.342–1.357)	1.268 (1.26–1.276)
≥ 30	168,440	1,061,409.6	37.4653	1.436 (1.422–1.451)	1.36 (1.342–1.378)
**Elevated waist circumference**^**b**^					
No	739,359	26,498,105.14	27.9023	1	1
Yes	122,481	3,382,420.03	36.2111	1.296 (1.288–1.304)	1.057 (1.048–1.065)
**Drink**					
None	311,253	11,517,820.22	27.0236	1	1
Mild	470,110	15,819,909.38	29.7164	1.099 (1.095–1.104)	1.04 (1.035–1.045)
Heavy	80,477	2,542,795.58	31.649	1.171 (1.162–1.18)	1.053 (1.044–1.061)
**Exercise**					
No	745,814	26,253,605.67	28.4081	1	1
Yes	116,026	3,626,919.5	31.9902	1.126 (1.119–1.133)	1.071 (1.064–1.077)

Values are expressed as hazard ratios (95% confidence intervals) using univariate and multivariate Cox proportional hazards regression analyses.

*BMI* body mass index, *CI* confidence interval, *HR* hazard ratio.

^a^Incidence rates are expressed in units of per 1000 person-years.

^b^Elevated waist circumference is defined as a waist circumference of more than 90 cm in men or more than 85 cm in women.

Multivariate analysis was then performed with variables that showed a statistically significant difference from the univariate analysis, including sex, age, BMI, waist circumference, drinking status, and exercise status. As shown in Table 2, all the variables included in the multivariate analysis were associated with an increased risk of dermatophytosis. Female sex tended to have a protective effect (HR 0.848, 95% CI 0.843–0.853) against dermatophyte infection. Age of older than 30 years (HR 1.064; 95% CI 1.059–1.068), having a higher BMI (HR 0.876; 95% CI 0.868–0.884 for those with BMIs < 18.5 kg/m^2^ vs. HR 1.153; 95% CI 1.146–1.159 for those with BMIs of 23–24.9 kg/m^2^ vs. HR 1.268; 95% CI 1.26–1.276 for those with BMIs of 25–29.9 kg/m^2^ vs. HR 1.36; 95% CI 1.342–1.378 for those with BMIs of ≥ 30 kg/m^2^), having a greater waist circumference (HR 1.057; 95% CI 1.048–1.065), being a heavier drinker (HR 1.04; 95% CI 1.035–1.045 for mild drinkers vs. HR 1.053; 95% CI 1.044–1.061 for heavy drinkers), and engaging in mild-to-heavy exercise (HR 1.071; 95% CI 1.064–1.077) were statistically significant predictors for developing a dermatophyte infection.

### Cumulative impact of BMI status on dermatophytosis incidence

**Figure 2:**
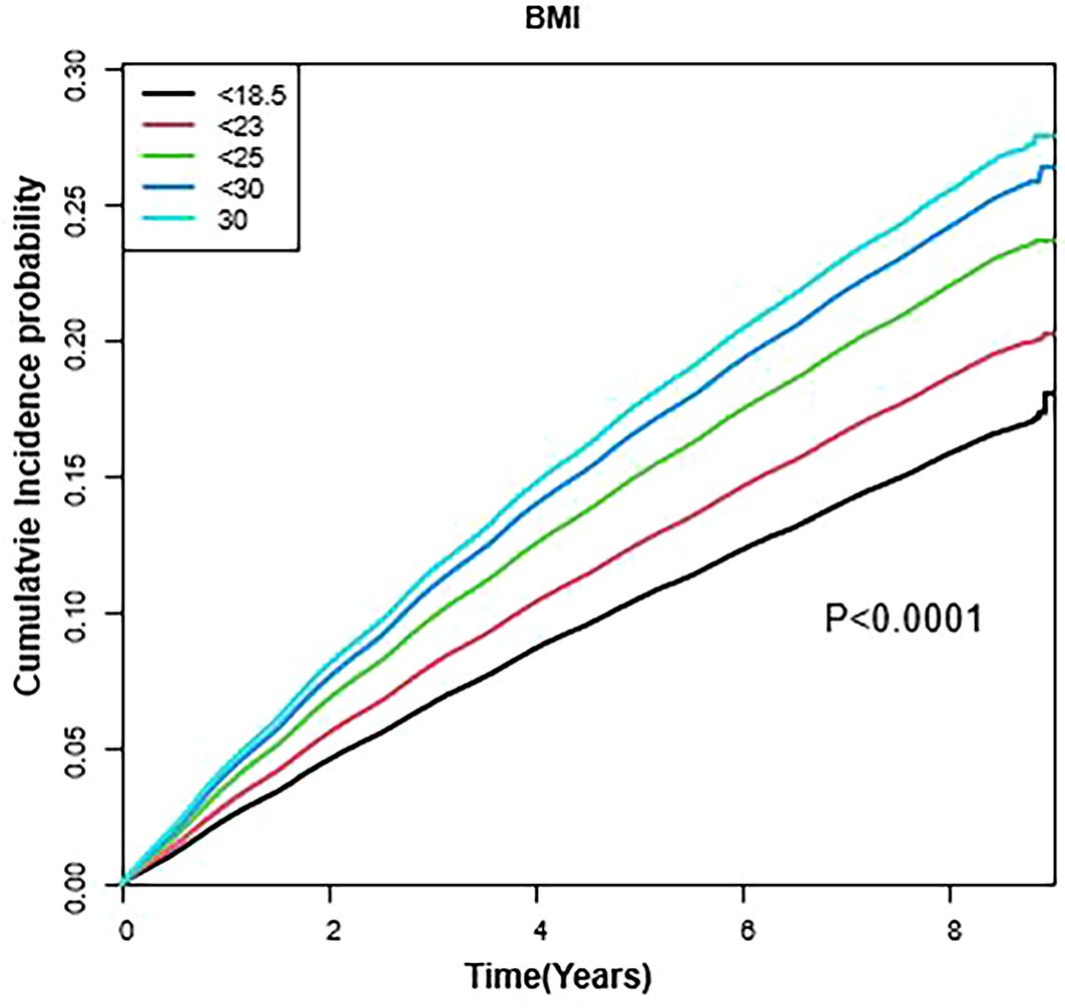
Cumulative incidence of dermatophytosis according to body mass index during the study period. *P*-values were determined using the log-rank test adjusted for age, sex, smoking status, alcohol consumption, physical activity, household income, diabetes mellitus, hypertension, and dyslipidemia.

The results of Kaplan–Meier analysis for the cumulative incidence rates of dermatophytosis based on the BMI status adjusted for sex, age, alcohol consumption, smoking status, physical activity, household income, diabetes mellitus, hypertension, and dyslipidemia are shown in Fig. 2. The cumulative incidence rates of dermatophytosis increased linearly and showed a steeper increase in higher BMI groups (*P* < 0.0001, log-rank test; Fig. 2.).

## Discussion

In this large-scale nationwide study of the Korean population, the data of 4,532,665 subjects aged between 20 and 40 years were analyzed to determine the risk factors associated with the development of dermatophytosis in Korea. Notably, women were less likely to be infected by dermatophytes than men, while those who were older and those with higher incomes; higher blood pressure values; higher low-density lipoprotein cholesterol levels; lower high-density lipoprotein cholesterol levels; and more comorbidities, such as diabetes, dyslipidemia, and hypertension, were determined to be more susceptible to dermatophytosis. Also, drinking more alcohol, performing exercise more frequently, having a history of smoking, and having a greater waist circumference also increased the risk of developing a dermatophyte infection, even after adjusting the confounding factors.

It has been widely reported that dermatophytosis is associated with male sex; a systemic or local immunocompromised status, such as diabetes mellitus; and long-term use of topical steroids^9,10^. Previous known risk factors of tinea pedis and onychomycosis, the most common types of tinea, accounting for 33% to 40% of all dermatophytosis, includes advanced age; male sex; nail trauma; peripheral vascular insufficiency; and immunosuppression status, such as diabetes mellitus and human immunodeficiency virus infection^9,11^. Warm and humid foot conditions secondary to wearing tight shoes; performing specific sporting activities, such as swimming; smoking; and obesity were also reported to increase the risk of tinea pedis and onychomycosis^11–13^. On the other hand, tinea capitis mainly occurs in children between the ages of three and 14 fours, and known risk factors include male sex, low socioeconomic status, overcrowding, and keeping pets at home^14,15^. Meanwhile, tinea capitis in the adult population is highly associated with comorbidities, such as rheumatoid arthritis, human immunodeficiency virus infection, diabetes mellitus, leukemia, and kidney failure^15^.

In this study, male sex, advanced age, obesity, alcohol consumption, and moderate to heavy exercise were shown as risk factors for the development of dermatophytosis. Among these risk factors, sex was the most influential variable. Tinea cruris is the most known sex-influenced dermatophytosis, found to be three times more common in men than in women^14^. In addition, men also have a higher risk of developing tinea pedis than women, which also may be due to their more common exposure to moist environments due to wearing of occlusive footwear and more frequent physical exercise^14^. These aforementioned lifestyle characteristics more commonly associated with male sex are thought to be responsible for the high prevalence of dermatophytosis in men; however, it is not yet understood whether male sex itself increases the susceptibility to infection^10,14^.

In our study, advanced age was shown to be the risk factor for developing dermatophytosis. Yoon et al.^4^ reported that the prevalence of dermatophytosis in Korea continuously increases with age, and the highest prevalence is among those aged between 60 and 69 years old. Among the types of dermatophytosis, onychomycosis, which is the most common superficial fungal infection, has been confirmed to increase with age^16^. Eleweski and Charif^17^ reported that approximately 40% of elderly patients have onychomycosis. The presumed cause for this high prevalence was to be that older individuals find it more difficult to exercise and care for feet and nails, increasing their susceptibility to colonization by infectious organisms^16,17^. Predisposing conditions, such as diabetes and compromised peripheral circulation, were also thought to be contributing causes^16,17^. These contributing factors, however, are thought to increase the prevalence of infection by all kinds of dermatophytes.

Obesity, expressed through the BMI index and waist circumference, was also found to be a significant contributing factor to the development of dermatophytosis. Onychomycosis has been relatively well studied regarding its relationship with obesity^18–20^. In a study of more than 1000 patients randomly screened to examine their feet, obesity was found to be one of the most prevalent predisposing factors among patients with fungal nail disease^18^. Onychomycosis was significantly increased in patients with obesity (odds ratio 2.13; 95% CI 1.45–3.13) in a study of 1245 diabetic Taiwanese patients^19^. In addition, topical antifungal treatment was shown to be less effective in patients who were overweight or obese^20^. Complete cure rates were 15.9% in obese patients and 22.0% in patients with healthy BMIs after 52 weeks of applying topical efinaconzole^20^. Several factors have been proposed to explain the potential mechanism behind the results. Firstly, obesity might make the skin more hospitable to fungal growth. Humid and warm conditions are important for fungal growth and survival. Thick layers of subcutaneous fat with deep skin folds may cause profuse sweating, and the trapped moisture and warmth may provide an optimal environment for the colonization of the dermatophytes^21,22^. Secondly, increased adipose tissue itself may further contribute to the increased risk for infection^23–25^. It has been recently recognized that adipose tissue participates actively in immunity through producing a variety of cytokines, such as leptin and adiponectin^24,25^. Leptin is a pro-inflammatory cytokine that activates polymorphonuclear neutrophils and T lymphocytes and regulates the activation of monocytes and macrophages^24,25^. However, obese patients often show leptin resistance, making them more vulnerable to infection^23^. Also, obese patients are more likely to have comorbidities, such as diabetes mellitus, which further contributes to the development of dermatophytosis^21,23^.

Lastly, engaging in routine exercise was also associated with an increased incidence of dermatophytosis. This is thought to be due to the wet environment caused by sweating from physical activities. However, as obesity is critical for the development of dermatophytosis and exercise is essential for maintaining a healthy weight, exercise should not be discouraged to manage the risk of developing dermatophytosis itself; instead, self-hygiene activities to keep the body clean and dry following each exercise session should be highlighted to reduce the risk of dermatophytosis after exercise.

This study also had some limitations. Firstly, dermatophytosis and comorbidities, such as hypertension, diabetes mellitus, and dyslipidemia, were identified using ICD-10 codes from claims databases. However, a validation study of the diagnostic codes of the KNHIS claims database revealed that only approximately 70% of the diagnosis codes coincided with those from medical records^26^. Secondly, dermatophytosis has some different clinical characteristics depending on the anatomic sites of infection, but this study did not distinguish these subtypes and was conducted while considering all subtypes of dermatophytosis as a whole. However, it is still meaningful to find out the risk factors for dermatophytosis as a whole without losing the significance since the fungi invading the skin share similar common characteristics. In contrast, the strengths of this study are its large sample size and nationally representative study population. Moreover, the national database contained information on socio-demographic characteristics, such as smoking status, alcohol consumption, physical activity, household income, and BMI, which were all found to be significant variables in the risk of developing a dermatophyte infection.

Taken together, our results demonstrated a significant positive association between the incidence of dermatophytosis and increasing BMI. In addition to male sex and increasing age, an elevated waist circumference, drinking, and exercise all contributed to the development of dermatophytosis. It is emphasized that lifestyle corrections directed at managing weight, drinking less alcohol, and keeping the body clean and dry after exercise might contribute to the prevention of dermatophytosis.

## Data Availability

The data that support the findings of this study are available from the Health Insurance Review & Assessment Service (HIRA). Restrictions apply to the availability of these data, which were used under license for the current study, and so are not publicly available due to personal information protection. Data are available at https://opendata.hira.or.kr/ with the permission of the HIRA.
